# Comparison of Transferred Deep Neural Networks in Ultrasonic Breast Masses Discrimination

**DOI:** 10.1155/2018/4605191

**Published:** 2018-06-21

**Authors:** Ting Xiao, Lei Liu, Kai Li, Wenjian Qin, Shaode Yu, Zhicheng Li

**Affiliations:** ^1^Shenzhen Institutes of Advanced Technology, Chinese Academy of Sciences, Shenzhen 518055, China; ^2^College of Communication Engineering, Chongqing University, Chongqing 400044, China; ^3^Department of Medical Ultrasonics, The Third Affiliated Hospital, Sun Yat-sen University, Guangdong 510630, China; ^4^University of Chinese Academy of Sciences, Beijing 100049, China

## Abstract

This research aims to address the problem of discriminating benign cysts from malignant masses in breast ultrasound (BUS) images based on Convolutional Neural Networks (CNNs). The biopsy-proven benchmarking dataset was built from 1422 patient cases containing a total of 2058 breast ultrasound masses, comprising 1370 benign and 688 malignant lesions. Three transferred models, InceptionV3, ResNet50, and Xception, a CNN model with three convolutional layers (CNN3), and traditional machine learning-based model with hand-crafted features were developed for differentiating benign and malignant tumors from BUS data. Cross-validation results have demonstrated that the transfer learning method outperformed the traditional machine learning model and the CNN3 model, where the transferred InceptionV3 achieved the best performance with an accuracy of 85.13% and an AUC of 0.91. Moreover, classification models based on deep features extracted from the transferred models were also built, where the model with combined features extracted from all three transferred models achieved the best performance with an accuracy of 89.44% and an AUC of 0.93 on an independent test set.

## 1. Introduction

Breast cancer is regarded as one of the high-incidence cancer types among women worldwide [1, 2]. Early detection of masses and nodules is crucial for successful treatment and reducing the mortality rate [2]. Ultrasonography is considered the most important adjunct method in clinical detection and diagnosis of breast cancer for its high availability, cost-effectiveness, acceptable diagnostic performance, and noninvasive and real-time capabilities [3].

As a valuable and beneficial means for breast cancer detection and classification, computer-aided diagnosis (CAD) system helps radiologists to detect and classify abnormalities like masses as either benign or malignant [4]. Current CAD system relies on multiple pipelines including preprocessing, tumor segmentation, feature extraction, feature selection, and machine learning-based classification [5]. Preprocessing is used to reduce speckle noise and facilitates segmentation, which aims to identify the surrounding tumors. Feature extraction is one of the most important steps in CAD system, followed by feature selection that reduces data dimension and improves model generalization. Most extracted features are explicitly designed or handcrafted, including tumor shape, intensity statistics, and texture features [6]. Based on a selected subset of features, a classifier can be built. The design of hand-crafted features significantly affects the classification performance.

Recently, Lingyun Cai et al. proposed a novel phase-based texture descriptor for a robust support vector machine (SVM) classifier to discriminate benign and malignant tumors in BUS images [7]. Similarly, Menon R V et al. adopted SVM method for classification through textural, morphological, and histogram feature metrics with principal component analysis (PCA) for dimension reduction [8]. In [9], a novel feature selection approach based on dual evaluation criteria was proposed to select 457 texture and shape features, with which Artificial Neural Network (ANN) and SVM were both used for classifying benign and malignant breast tumors. In general, current approaches mostly rely on manually designed features and a traditional classifier (such as AdaBoost [10] and SVM [11]) for masses type prediction. Although the number of handcrafted features has reached tens of thousands, these features are shallow and of low order, which may not fully characterize the heterogeneous pattern within the tumor. Moreover, extracting domain-specific image features extremely depends on a good understanding of the tumor in the radiological level. On the other hand, most previous studies require tedious operations like extensive preprocessing, image normalization, and lesion segmentation, which may significantly affect the repeatability of the classification method.

Deep learning algorithm, in particular Convolutional Neural Network (CNN), has been widely recognized as a reliable approach to learn predictive features directly from original images [12]. Many deep CNN models are presented for object detection and classification such as ResNet [13], InceptionV3 [14], and Xception [15]. The ResNet model [13] won the ImageNet Large Scale Visual Recognition Challenge (ILSVRC) [16] in 2015 with an error rate of 3.6%, outperforming human level (5%-10%) incredibly. Xception [15], proposed in 2016, is an extension of the inception architecture, which performs slightly better than InceptionV3 [14] in the ImageNet dataset. At present, deep CNN has become popular in the field of computer vision, as well as in the community of medical imaging analysis. For breast ultrasound image classification, several studies have been proposed [12, 17].

Although deep CNNs have been shown to be efficient classifiers, they always require a large amount of training data, which can be a difficult task for medical imaging data. When the target dataset is significantly smaller than the base dataset, transfer learning is believed to be a powerful tool for training deeper networks without overfitting [18]. In transfer learning, the training is performed in a two-step way that involves pretraining a deep neural network on a large dataset followed by a fine-tuning step by means of freezing the layers up to several convolutional blocks on a small local dataset. However, few studies have been done on developing and comparing transfer learning-based models for discriminating benign cysts from malignant masses in breast ultrasound images.

In this study, we proposed and compared five different models for classification of benign and malignant masses in BUS images. The five proposed models were a three-layer CNN model trained from scratch, a traditional classification model with hand-crafted features, and three transfer learning models built with pretrained CNN models: ResNet50, InceptionV3, and Xception. Moreover, a deep feature-combining model was built with an ANN model and deep features extracted from the above three transfer learning models. The contributions of our study are summarized as follows. (1) Instead of training specific CNNs from scratch, the proposed transfer learning method was able to learn effective features from the training data and achieved automatic classification of ultrasonic breast masses. (2) The transfer learning method outperformed the traditional machine learning model and the CNN model, while the deep feature-combining model achieved an improved performance compared to all the other tested classification models.

## 2. Materials and Methods

### 2.1. Convolutional Neural Network

**Figure 1:**
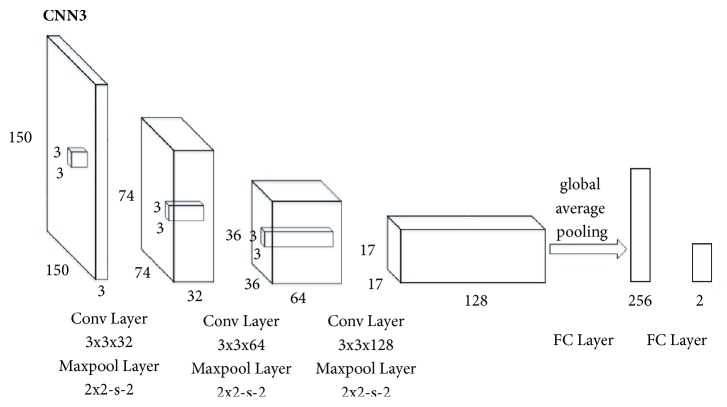
The architecture of CNN3. The network has 3 convolutional layers followed by 2 fully connected layers. 32-64-128-256-2 is the number of feature maps generated in each layer. 74-36-17 is the size of the feature maps. Global average pooling is used to reduce the total number of parameters. There are 256 neurons in the first fully connected layer and 2 neurons in the output layer indicating class scores of benign and malignant masses.

In the case of lacking enough samples to train deep neural networks, a shallow CNN model was designed.  Figure 1 illustrated the overall architecture of the CNN model (denoted CNN3) used in the paper. The breast ultrasound images were resized into 150×150 as the input of CNN3. Our architecture, CNN3, was made up of three convolutional layers and two fully connected layers with a softmax classification function. The number of model layers was experimentally determined in order to design a CNN model with optimized classification performance. All convolutional layers had 3×3 kernels stacked together with Rectified Linear Units (ReLUs) between each other followed by maxpooling layers with a stride of two. Particularly, the CNN3 model used global average pooling, which averages out the channel values across the 2D feature map after the last convolutional layer in order to reduce the total number of parameters. The two neurons in the output layer indicated class scores of benign and malignant masses.

### 2.2. Transfer Learning

When training dataset is relatively small, transferring a network pretrained on a large annotation dataset and fine-tuning it for a particular task are an efficient way to achieve acceptable accuracy and less training time [18]. Although classification of breast masses in BUS images differs from object recognition in natural images, they may share similar learned features [19]. It is expected that the deep features learned from top performing networks in the ILSVRC would also perform well in different task. Next, we will introduce three popular deep neural networks used for transfer learning in our study: ResNet50, InceptionV3, and Xception.

The ResNet model consists of a stack of similar (so-called residual) blocks, with each block being in turn a stack of convolutional layers [13]. The output of a block is also connected with its own input through an identity mapping path. This design alleviates the vanishing gradient problem and improves the gradient backward flow in the network, thus allowing training much deeper networks.

InceptionV3 [14] is a rethinking for the initial structure of InceptionV1 [20] and InceptionV2 [21]. The model is trained on the ImageNet dataset, which can identify 1000 classes with a top 5 error rate of 3.5% and top 1 error rate down to 17.3%. In addition, InceptionV3 manages memory more efficiently than other CNN models.

Xception [15] is based on the assumption that the correlation between the input channels is completely separable from the spatial correlation. Specifically, Xception extends the inception architecture by replacing standard convolution with depthwise independent convolution. It is a linear stack of deep collapsible layers with residual connections. Xception performs slightly better than InceptionV3 on the ImageNet dataset and outperforms much on a larger scale image dataset with 17000 categories using same number of parameters.

In the field of computer vision, many deep CNN architectures have been well trained for object detection and classification, and the models mentioned above are publicly available. Therefore, there is no need to train those deep neural networks from scratch [22]. We used the Keras module built on top of TensorFlow as the deep learning framework, where most top performing pretrained model weights were provided. Our approach included a two-step training process: (1) leveraging a network pretrained on a large dataset in source domain, which shares general features for most computer vision problems, and then (2) fine-tuning it on a small-scale local dataset in target domain by means of freezing the layers up to several convolutional blocks.

**Figure 2:**
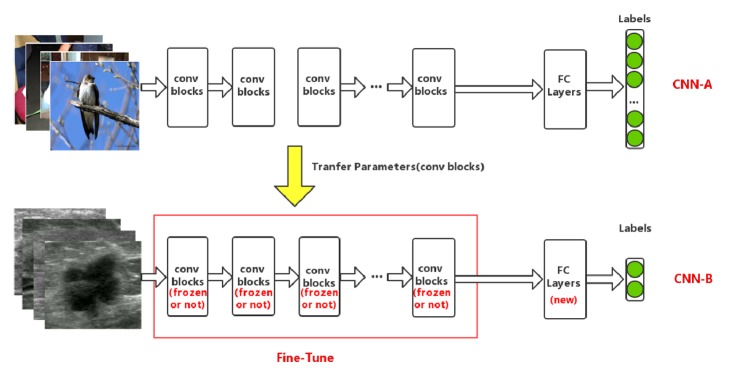
Overview of transfer learning framework in our paper.** Top row**: the CNN-A is pretrained on the ImageNet database for classification, which consists of many convolutional blocks and fully connected layers.** Bottom row**: after modifying the structure of fully connected layers, the CNN-B model (except fully connected layers) is initialized with the previous trained weights from CNN-A, the first* n* convolutional blocks of which are locked, while the left are unlocked. Then the entire network is trained on breast ultrasound images to fine-tune the remaining unlocked layers.

The transfer learning framework used in this paper is illustrated schematically in Figure 2. For example, when InceptionV3 was selected as the base CNN (denoted as CNN-A), specific operation was described as follows. First, to adapt to the target domain, the number of the fully connected layers and neurons in each layer was modified accordingly. Then a new network model, CNN-B, was obtained. In addition, only the convolutional layers of CNN-B were instantiated with weights of the CNN-A model pretrained on the ImageNet dataset. Finally, to improve the classification performance, the parameters of the last several convolutional blocks of the InceptionV3 model were fine-tuned on our own BUS dataset.

### 2.3. Feature Combination

**Figure 3:**
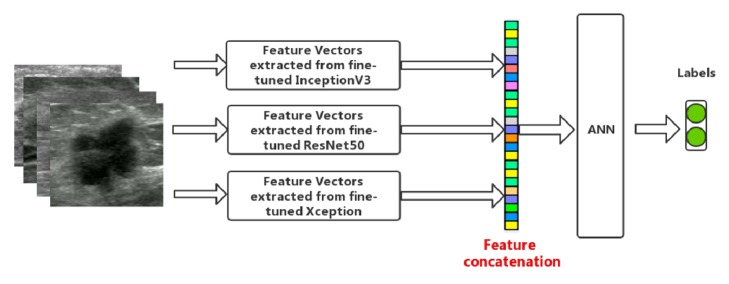
Illustration of feature combination conducted in this paper.

Figure 3 illustrates the feature-combining model. The above three pretrained deep neural network models were fine-tuned on our BUS dataset firstly. Then features were extracted and combined by means of feature concatenation. Finally, ANN was adopted for classification of breast masses. Note that different combination of the three groups of deep features was used for classification.

## 3. Experiments

Experiments were conducted to evaluate the performance of five models on breast masses classification, including a traditional machine learning-based model, a CNN3 model, and three transfer learning models. Finally, a deep feature-combining model was built with features extracted from the above three fine-tuned pretrained deep CNNs, where an ANN was used for performance evaluation and comparison in classifying breast masses. Experiments were based on a 64-bit Ubuntu 16.04 operating system with a 32GB memory and a NVIDIA GTX1080 GPU.

### 3.1. Data

**Figure 4:**
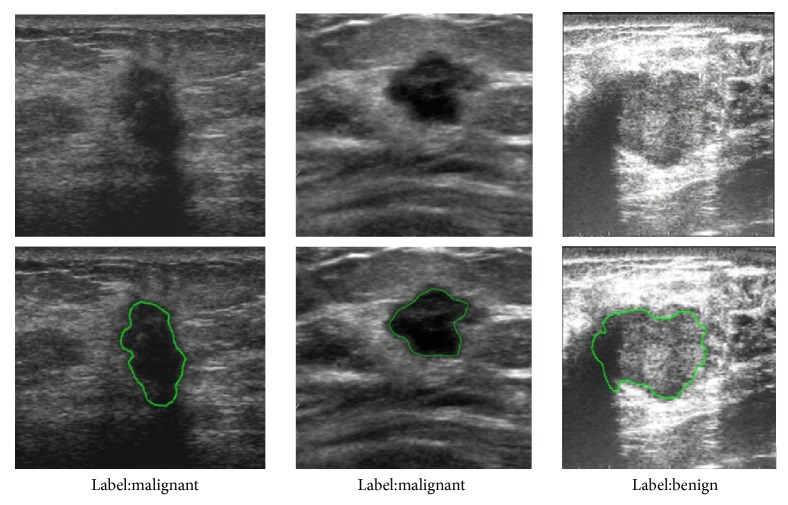
Three representative breast masses.

In this retrospective study, a cohort of 1422 patients was collected from the Third Affiliated Hospital of Sun Yat-sen University between 2014 and 2017. In total, 2058 masses were observed and used for building and validating the models: 688 malignant solid masses and 1370 benign masses. All masses were confirmed by tissue samples obtained via biopsy or operation. The contours of the masses were manually delineated by an experienced radiologist.  Figure 4 shows three representative cases, each of which is provided with annotation of category label and lesion contours.

During training, standard data augmentations such as rescale, flip, and zoom were applied. However, we did not apply rotation to the images tagged as "habit," as it may change some key diagnostic properties of breast masses like aspect ratio. The converted images were resized to meet requirement of specific models. For ResNet50, Xception, and InceptionV3 models, the input image was resized to 224×224, 299×299, and 299×299, respectively.

### 3.2. Implementation Details

In the traditional model, the lesion contours of breast ultrasound images were segmented by an experienced radiologist. Within the segmented mass, we extracted various hand-crafted features for model building, including 18 first-order features, 12 texture features, and 8 morphological features (listed in Table 1). We built the classifier using both AdaBoost and SVM. Before classification, feature selection was required to reduce the data dimension. Here we employed the Linear Discriminant Analysis (LDA) as the feature selection method.

For CNN3 and three transfer learning models, Batch normalization [21] was employed to speed up the training of fully connected layers. Dropout [23] was applied with P = 0.5. The probability of each image sample belonging to the malignant or benign mass was computed with a softmax classifier. We used the rectified liner unit activation function in each layer. The objective function used was categorical cross-entropy. And the model was trained using Adam with a batch size of 16 as learning rule. Additionally, in the process of transfer learning, three fully connected layers were added with {1024, 512, 2} units.

10-fold cross-validation was used to assess the traditional model, CNN3, and the transfer learning models, where all BUS images were split into two parts, training set (90%) and validation set (10%), during each round of validation. Training set was used to train the model, while the performance of each model was evaluated on the validation set. Specifically, there were 1852 masses (1233 benign masses and 619 malignant masses) in the training set and 206 masses (137 benign masses and 69 malignant masses) in the validation set.

In the deep feature-combining models, we randomly split the BUS images into three parts, namely, training set (80%), validation set (10%), and test set (10%). Training set was used to train the model, while the validation set was used for selecting the model with the smallest error. The test set was used for independent performance evaluation. The ANN classifier containing three-layer neural networks with a 1024-512-2 architecture was trained using the Adam algorithm.

### 3.3. Performance Evaluation Criteria

In our study, the classes (benign and malignant mass) were not equally represented. This imbalance may cause poor classification accuracy for the minority class [24]. To comprehensively evaluate the classification performance on the imbalanced dataset, the accuracy, sensitivity, specificity, receiver operating characteristic (ROC) curve, precision recall (PR) curve, and F1 score were calculated. The sensitivity, specificity, accuracy, and F1 score can be calculated as

(1) $$\begin{array}{c} specificity=\frac{TN}{TN+FP} \end{array}$$

(2) sensitivity=TPTP+FN

(3) accuracy=TP+TNTP+TN+FN+FP

(4) F1=2TP2TP+FN+FP

TP is the number of correctly predicted malignant lesions, while FP is the number of mistakenly predicted ones. Likewise, TN represents the number of correctly predicted benign lesions, and FN represents the number of mistakenly predicted omes. Based on the ROC curve, the area under ROC curve (AUC) was also calculated.

## 4. Results

Table 2 summarizes the performance of traditional machine learning model in breast masses classification. In terms of classification accuracy, the effect of morphological features (70.41%) was better than texture features (66.52%) and first-order features (67.35%). Through experimental analysis, the combined morphological features and texture features used in AdaBoost classifier can achieve an accuracy of 69.53%, sensitivity of 55.42%, and specificity of 74.85%. When combining all the features above, an accuracy of 69.67%, sensitivity of 55.57%, and specificity of 75.13% were achieved using AdaBoost classifier. It can be also observed that both AdaBoost and SVM classifiers with LDA feature selection achieved improved performance.

The performance of the CNN3 model directly learned from our local ultrasound data is also shown in Table 2. Compared with all tested traditional models, the CNN3 model achieved the highest performance in terms of accuracy (74.44%), sensitivity (63.19%), specificity (79.22%), AUC (0.78), and F1 score (0.60).

The classification performance of the three transferred deep neural networks is displayed in Table 3. These models are InceptionV3, ResNet50, and Xception. It can be found that transfer learning model with pretrained InceptionV3 network achieved the top performance with the highest accuracy of 85.13%, AUC of 0.91, and F1 score of 0.78. The accuracy rates of ResNet50 and Xception models were slightly degraded to 84.94% and 84.06%, respectively. From Tables 2 and 3, it is observed that the transferred InceptionV3 model achieved the best accuracy among all five compared models. The specificity reflects the diagnostic ability to exclude benign breast cancers, while sensitivity reflects the ability to detect malignant breast cancers. It is shown in Table 3 that transferring InceptionV3 model on our own BUS dataset achieved the highest sensitivity (77.44%) and specificity (89.06%) among all tested models.

**Figure 5:**
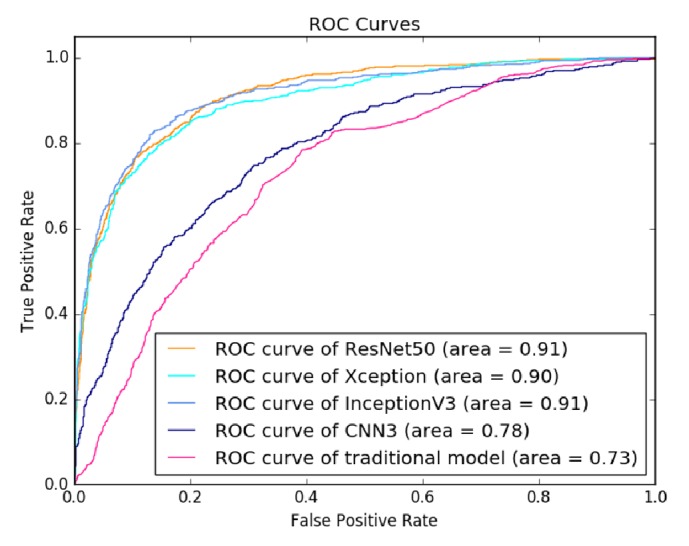
ROC curves of evaluated classification models on BUS dataset.

**Figure 6:**
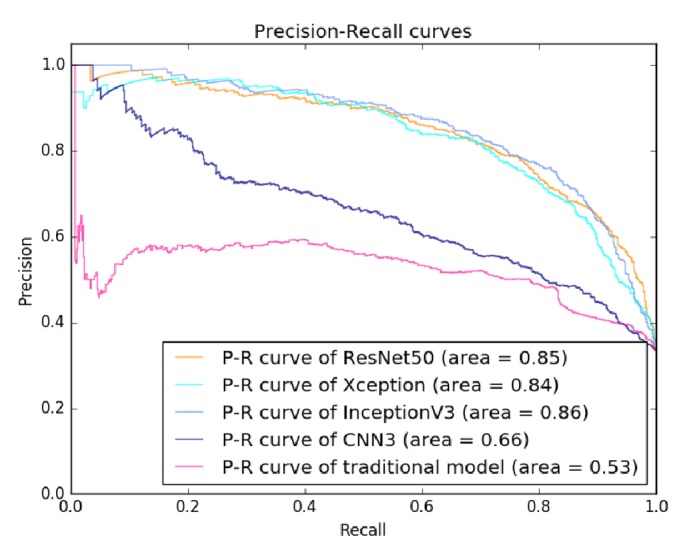
Precision-recall curves of evaluated classification models on BUS dataset.

Figure 5 shows the ROC curves of all tested models. The transferred InceptionV3 and ResNet50 models achieved an equal AUC of 0.91, while the AUC of the transferred Xception model was slightly lower (0.90). Moreover, the AUC of the transfer learning models significantly outperformed both the CNN3 (0.78) and the traditional model (0.73).  Figure 6 shows the PR curves of the tested models. It can be also observed that the transfer learning models significantly outperformed the CNN3 and the traditional model, where the transferred InceptionV3 model achieved the best performance among all the models.

**Figure 7:**
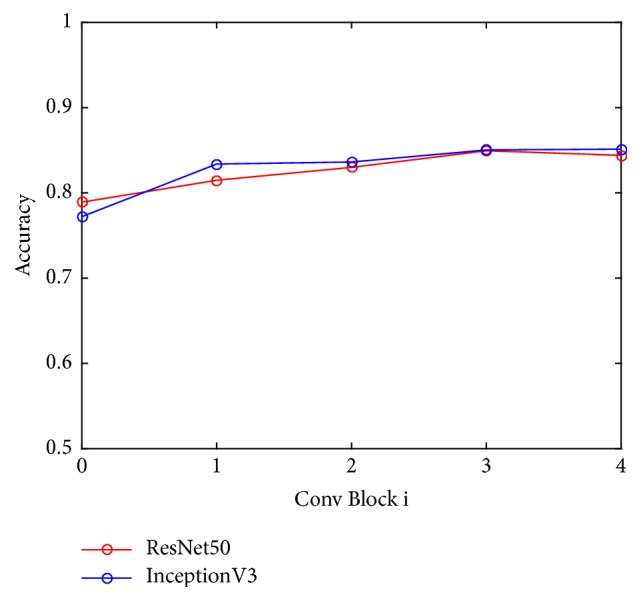
Classification performance versus the number of fine-tuned convolutional blocks (Conv block 0 indicates the performance without fine-tuning convolutional blocks).

Furthermore, Figure 7 indicates how the number of fine-tuned convolutional blocks influenced the classification performance. For all tested deep neural models, fine-tuning the last convolutional blocks can improve the performance compared with keeping the weights of all the convolutional layers fixed. For example, fine-tuning the last convolutional block of ResNet50 network achieved an accuracy of 81.48%. However, the performance does not solely depend on how deep the base model is fine-tuned. There was a decrease in classification accuracy when fine-tuning more than three convolutional blocks for ResNet50 model.

The classification performance of the deep feature-combining model is summarized in Table 4. It can be found that the model built with combined deep features extracted from all three transferred models achieved the best performance in terms of accuracy (89.44%), sensitivity (88.73%), specificity (89.91%), AUC (0.93), and F1 score (0.87). Generally, the models built with deep features from two transferred models were better than those built with features from only one model.

**Figure 8:**
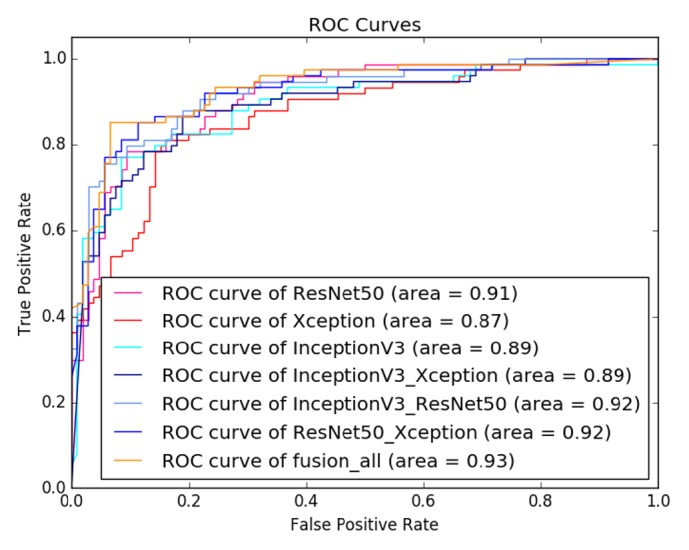
ROC curves of evaluated models with feature combination on BUS dataset.

**Figure 9:**
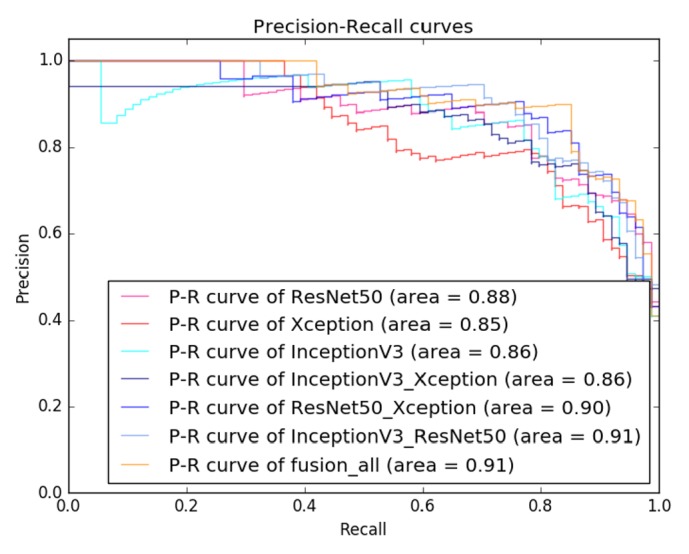
Precision-recall curves of evaluated models with feature combination on BUS dataset.

Figures 8 and 9 are the ROC curves and PR curves for all tested deep feature-combining models, respectively. It can be found that the model built with features extracted from all the three transferred models achieved the best overall performance.

## 5. Discussion

The main finding of this study was that the transferred CNN models outperformed both the CNN trained from scratch and the traditional model, while the deep feature-combining model achieved the best performance for classification of benign and malignant breast masses from ultrasound images. Traditional models were built with hand-crafted image features and a machine learning-based classifier. The extraction of domain-specific imaging features largely depends on the designer's prior knowledge. Our experiments show that the classification problem can be well addressed by using transferred CNN models, which were able to learn effective features based on the pretrained models and achieved better performance in breast masses classification.

Our study investigated the technique of transfer learning that fine-tuned the deep neural network models pretrained on large-scale natural image dataset. According to Table 3 and Figures 5 and 6, the proposed approach performed well in breast masses classification by transferring three CNN models (InceptionV3, ResNet50, and Xception). Among the three models, the transferred InceptionV3 achieved the best accuracy. Our result demonstrated that transferring InceptionV3 model pretrained on natural image dataset could be an effective way to build deep neural network model for classification of breast masses in medical ultrasonic images.

It is revealed in the experiments that CNNs initialized with large-scale pretrained networks outperformed those directly learnt from small-scale ultrasound data with accuracy improvements of 7% to 11%. This can be explained by the fact that the CNN model cannot learn the true data distribution from a small dataset and therefore is likely to overfit the training data. Thus, with small-scale ultrasound image dataset, we suggest the use of transferred CNN models for classification of breast masses rather than learning a deep neural network from scratch.

Our results also indicated that there was a trade-off between the number of fine-tuned convolutional blocks and the classification accuracy as shown in Figure 7. Therefore, it is promising to apply transfer learning with a balance between the scale of image dataset and the complexity of CNN models. In fact, features learned from pretrained deep neural models on a large natural image dataset without fine-tuning could be specific to natural images, which may not generalize well in medical images. When fine-tuning certain convolutional blocks, the model was further generalized on BUS dataset by learning new representative features. Thus, the model was capable of classifying masses in BUS images. When the depth of the fine-tuned convolutional blocks exceeds a certain number, the deep network model may not be well trained based on the small-scale image samples in our BUS data. In such a case, overfitting was prone to occur, resulting in a decrease in classification accuracy.

From Table 4 and Figures 8 and 9, the deep feature-combining model built with features extracted from the three fine-tuned CNN models (ResNet50, InceptionV3, and Xception) achieved the highest accuracy. These three models were pretrained on a large-scale dataset, so we believe feature derived from these models can fully characterize the image heterogeneity, which is of essential importance for classification of tumor types. Combination of features extracted from multiple deep convolutional models can capture more image patterns, which may be useful for identifying malignant breast masses.

## 6. Conclusion

In this paper, we proposed and compared five different models for classification of benign and malignant masses in BUS images. The five proposed models are a CNN model trained from scratch, a traditional classification model with hand-crafted features, and three transfer learning models built with pretrained CNN models: ResNet50, InceptionV3, and Xception. Finally, a deep feature-combining model was built with an ANN model and deep features extracted from the above three transfer learning models. Among the CNN models discussed in this paper, transferred InceptionV3 achieved the best results on our own BUS dataset with an accuracy of 85.13% and an AUC of 0.91, outperforming not only traditional machine learning models but also the CNN3 model directly learnt from small-scale ultrasound data. Transferring InceptionV3 model pretrained on a large-scale natural image dataset could be an effective way to build deep neural network model for classification of breast masses on a small-scale ultrasonic image dataset. Additionally, combining transferred features from multiple CNNs could further improve the classification accuracy.

In future work, with a larger BUS image dataset, we can exploit and design specific neural networks for tumor classification. In addition, it should be noted that although transferred InceptionV3 achieved a better performance, it is memory-consuming and therefore may not be suitable for embedded devices. For embedded devices, some more memory-saving models such as shallower architectures might be a better choice.

## Figures and Tables

**Table 1:** Features extracted in traditional model.

Feature category	Feature description
First-order features	FT1: Energy, FT2: Entropy, FT3: Minimum, FT4: 10th percentile, FT5: 90th percentile, FT6: Maximum, FT7: Mean, FT8: Median, FT9: Interquartile Range, FT10: Range, FT11: Mean Absolute Deviation (MAD), FT12: Robust Mean Absolute Deviation (rMAD), FT13: Root Mean Square (RMS), FT14: Standard Deviation, FT15: Skewness, FT16: Kurtosis, FT17: Variance, FT18: Uniformity

Texture features	Global texture features: FM1: Variance, FM2: Skewness, FM3: Kurtosis Gray-Level Cooccurrence Matrix (GLCM) texture features: FM4: Energy, FM5: Contrast, FM6: Correlation, FM7: Homogeneity, FM8: Variance, FM9: Sum Average, FM10: Entropy, FM11: Dissimilarity, FM12: Autocorrelation

Morphological features	FB1: Circularity, FB2: Elongation, FB3: Compactness, FB4: Roughness, FB5: Orientation, FB6: Radial distance standard deviation, FB7: Maximum chord length, FB8: Second moment

**Table 2:** Quantitative classification results based on traditional approaches and CNN model.

Model	Specificity	Sensitivity	Accuracy	AUC	F1
First-order features + AdaBoost	71.00%	52.02%	67.35%	0.66	0.38

Texture features + SVM	66.80%	48.69%	66.52%	0.52	0.04

Morphological features + AdaBoost	75.18%	57.22%	70.41%	0.72	0.51

First-order features + Morphological features + AdaBoost	74.73%	54.95%	69.29%	0.73	0.49

Texture features + First-order features + AdaBoost	70.38%	49.87%	66.52%	0.65	0.36

Texture features + Morphological features + AdaBoost	74.85%	55.42%	69.53%	0.72	0.50

Texture features + Morphological features + First-order features + AdaBoost	75.13%	55.57%	69.67%	0.72	0.50

Texture features + Morphological features + First-order features + AdaBoost with LDA	74.61%	58.10%	70.55%	0.73	0.49

Texture features + Morphological features + First-order features + SVM	66.93%	37.50%	64.53%	0.53	0.15

Texture features + Morphological features + First-order features + SVM with LDA	77.00%	58.96%	71.77%	0.68	0.55

**CNN3**	**79.22%**	**63.19%**	**74.44%**	**0.78**	**0.60**

**Table 3:** Comparison of quantitative classification results based on different models.

Model	Specificity	Sensitivity	Accuracy	AUC	F1
ResNet50	88.74%	77.39%	84.94%	0.91	0.78

Xception	87.16%	77.44%	84.06%	0.90	0.76

**InceptionV3**	**89.06%**	**77.44%**	**85.13%**	**0.91**	**0.78**

CNN3	79.22%	63.19%	74.44%	0.78	0.60

Traditional model	74.61%	58.10%	70.55%	0.73	0.49

**Table 4:** Comparison of quantitative classification results with feature combination.

Features	Specificity	Sensitivity	Accuracy	AUC	F1
Transferred features based on ResNet50	83.05%	87.10%	84.44%	0.91	0.79

Transferred features based on Xception	80.53%	77.61%	79.44%	0.87	0.74

Transferred features based on InceptionV3	82.20%	85.48%	83.33%	0.89	0.78

Transferred features based on InceptionV3 and Xception	80.99%	86.44%	82.78%	0.89	0.77

Transferred features based on ResNet50 and InceptionV3	86.49%	85.51%	86.11%	0.92	0.83

Transferred features based on ResNet50 and Xception	87.39%	86.96%	87.22%	0.92	0.84

**Transferred features based on ResNet50, Xception, and InceptionV3**	**89.91%**	**88.73%**	**89.44%**	**0.93**	**0.87**

## Data Availability

Breast ultrasound images in this research were acquired directly from the Third Affiliated Hospital of Sun Yat-sen University. And the diagnostic data captured is from patients from 2014 till now. It consists of 2058 cases with 688 malignant solid masses and 1370 benign masses. All the diagnosis results of the cases were confirmed by both biopsy and operation with high credibility. Meanwhile, all tumors were annotated by an experienced reader.
